# Primary Percutaneous Coronary Intervention in Patients With Type 2 Diabetes With Late/Very Late Stent Thrombosis and *de novo* Lesions: A Single-Center Observational Cohort Study of Clinical Outcomes and Influencing Factors

**DOI:** 10.3389/fcvm.2021.653467

**Published:** 2021-06-22

**Authors:** Xiaoxiao Zhao, Jun Lan, Xiaoping Yu, Jinying Zhou, Yu Tan, Zhaoxue Sheng, Jiannan Li, Ying Wang, Runzhen Chen, Chen Liu, Peng Zhou, Yi Chen, Li Song, Hanjun Zhao, Hongbing Yan

**Affiliations:** ^1^Department of Cardiology, Fuwai Hospital, National Center for Cardiovascular Diseases, Beijing, China; ^2^Department of Cardiovascular Medicine and Dongguan Cardiovascular Institute, Songshan Lake (SSL) Central Hospital of Dongguan City, The Third People's Hospital of Dongguan City, Affiliated Dongguan Shilong People's Hospital of Southern Medical University, Dongguan, China; ^3^Fuwai Hospital Chinese Academy of Medical Sciences, Shenzhen, China; ^4^Peking Union Medical College and Chinese Academy of Medical Sciences, Xiamen University, Fujian, China

**Keywords:** *de novo* lesions, late or very late stent thrombosis, diabetes mellitus, percutaneous coronary intervention, metabolic

## Abstract

**Background:** This study compared differences in the risk factors and clinical outcomes of primary percutaneous coronary intervention (PCI) in type 2 diabetes mellitus (DM) and non-DM patients with *de novo* lesions (DNLs) and late or very late stent thrombosis (LST/VLST).

**Methods:** We used angiography to screen 4,151 patients with acute coronary syndrome for DNL and LST/VLST lesions. Overall, 3,941 patients were included in the analysis and were allocated to the DM (*n* = 1,286) or non-DM (*n* = 2,665) group at admission. The primary endpoint was a composite of major adverse cardiovascular events (MACEs), defined as death, myocardial infarction, revascularization, and ischemic stroke, within a median follow-up period of 698 days.

**Results:** In the group with a total white blood cell count >10 × 10^9^/L (*P* = 0.004), a neutral granular cell count >7 × 10^9^/L (*P* = 0.030), and neutrophil–lymphocyte ratio >1.5 (*P* = 0.041), revascularization was better for DNL than for LST/VLST lesions. Among DM patients with DNLs, each unit increase in age was associated with a 53.6% increase in the risk of MACEs [hazard ratio (HR): 1.536, 95% confidence interval (CI), 1.300–1.815, *P* < 0.0001]. Older age (≥65 years) was associated with a significantly greater risk of MACEs (*P* < 0.0001). Furthermore, each standard deviation (SD) increase in the level of peak white blood cell counts was associated with a 50.1% increase in the risk of MACEs (HR, 1.501; 95% CI, 1.208–1.864; *P* = 0.0002). When stratifying the DM population with DNLs according to the D-dimer baseline and peak levels <0.5 vs. ≥0.5 mg/L, the high D-dimer group at baseline had a 2.066-fold higher risk of MACEs (*P* < 0.0001), and the high peak level D-dimer group had a 1.877-fold higher risk of MACEs (*P* = 0.001) compared to the low-level groups. Among DM patients with LST/VLST, each unit increase in age was associated with a 75.9% increase in the risk of MACEs (HR: 1.759, 95% CI, 1.052–2.940, *P* = 0.032). Furthermore, for each SD increase in the peak D-dimer level, the risk of MACEs increased by 59.7% (HR, 1.597; 95% CI, 1.110–2.295; *P* = 0.041).

**Conclusion:** Following successful primary PCI, the measurement of baseline and peak D-dimer values may help identify individuals at high cardiovascular risk. This suggests a potential benefit of lowering D-dimer levels among T2DM patients with DNL. Furthermore, age and the peak D-dimer values may facilitate the risk stratification of T2DM patients with LST/VLST.

## Introduction

Patients with type 2 diabetes mellitus (DM), a pro-inflammatory disease (1), exhibit an enhanced inflammatory reaction at the site of implantation of stents. Compared with non-DM patients, DM patients who have undergone stent implantation often present neointimal hyperplasia and diffusely diseased vessels, along with deleterious local phenomena (2), various healing responses, and arterial remodeling (3). However, the long-term prognosis of DM patients with *de novo* lesions (DNLs) and very late stent thrombosis (LST/VLST) who have undergone primary percutaneous coronary intervention (PCI) remains unknown.

This retrospective, single-center, all-comer trial aimed to compare the differences in the long-term prognosis of DM and non-DM patients who underwent PCI for DNLs and for LST/VLST.

## Methods

### Study Population and Design

This retrospective observational study adhered to the Strengthening the Reporting of Observational Studies in Epidemiology statement. This study was conducted according to the principles outlined in the Declaration of Helsinki and was approved by the Ethics Committee of Fuwai Hospital. All study subjects provided written informed consent.

**Figure 1:**
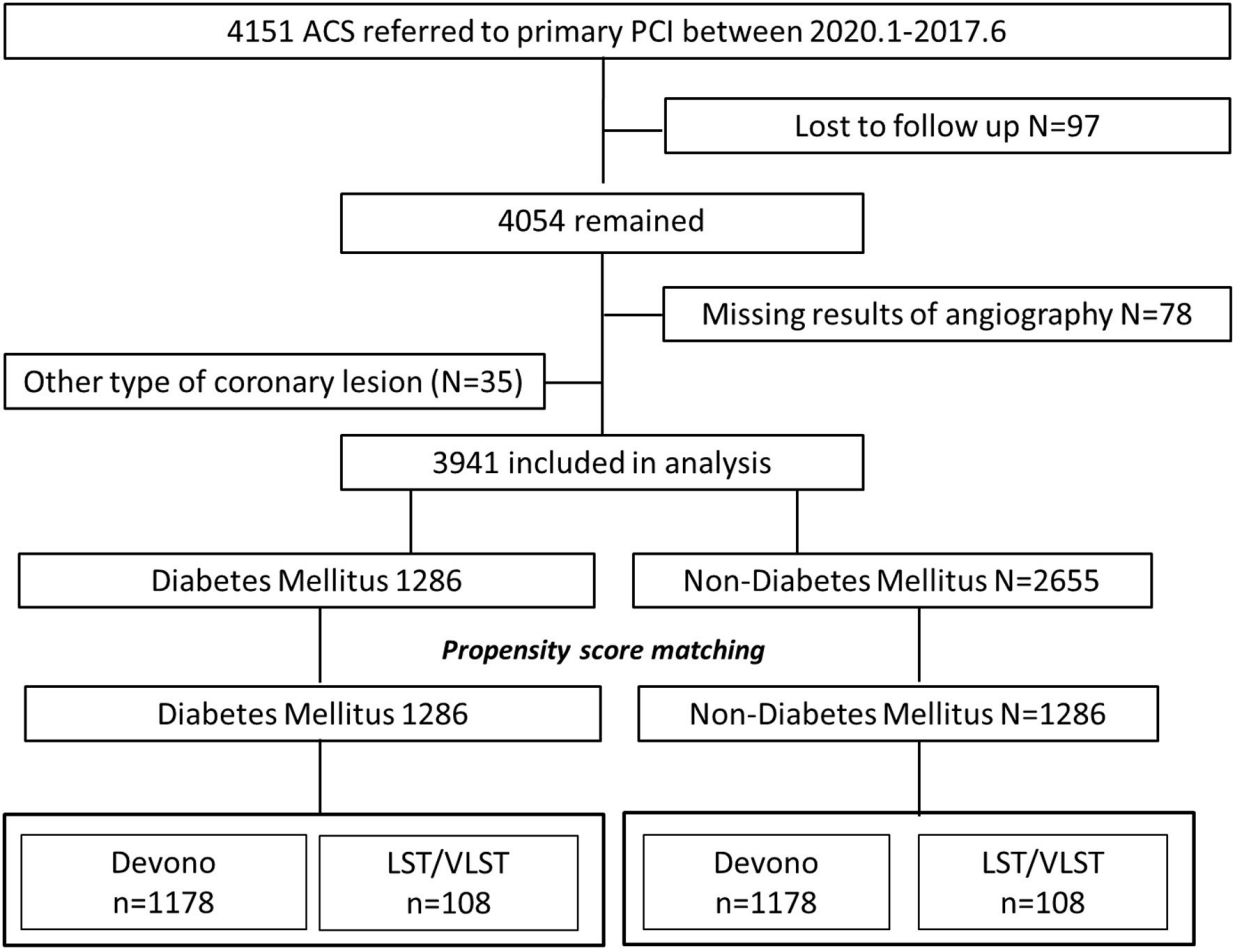
Flow chart. ACS, acute coronary syndrome; LST, late stent thrombosis; VLST, very late stent thrombosis.

The study was conducted on patients who had undergone primary PCI at Fuwai Hospital (National Center for Cardiovascular Diseases, Peking Union Medical College and Chinese Academy of Medical Sciences) in Beijing, China, between January 2010 and June 2017. From among 4,151 patients admitted for acute myocardial infarction (MI), 3,941 patients were included in this study (Figure 1) and were divided into a DM group (*n* = 1,286) and a non-DM group (*n* = 2,655). The types of coronary lesions, including DNL (*n* = 3,661) and LST/VLST (*n* = 280), were identified angiographically. Patients who were lost to follow-up, whose coronary angiography parameters were not available, or who refused participation were excluded from the analysis.

By using coronary angiography, stent thrombosis (ST) was defined as when a thrombus originated in a segment 5 mm distal or proximal to the stent, or in the stent, in patients with acute coronary syndrome (ACS) (4). The Academic Research Consortium (ARC) defined late ST as ST that occurred between 30 days and 1 year, and VLST as ST that occurred >1 year, after stent implantation (4). Three independent and blinded interventional cardiologists with >5 years' experience in interventional cardiology screened all patients with a history of stent implantation. Anonymized angiographic data for each patient were allocated to two of the three cardiologists at random. The cardiologists analyzed the data independently and blindly; disagreements were resolved by discussion to consensus among all three cardiologists.

### Clinical Outcomes

Clinical outcomes were obtained during follow-up via a telephone call or were confirmed from health records, as approved by the Review Board of Fuwai Hospital. The primary endpoints were major adverse cardiovascular events (MACEs), all-cause death, cardiac-related death, recurrent MI, revascularization, and ischemic stroke. A MACE was a composite of all-cause death, recurrent MI, and ischemic stroke. The physicians in charge of the follow-up identified and extracted primary endpoints from hospital records, laboratory reports, and clinical notes in the event of death.

### Statistical Analyses

Time-to-event variables are presented as Kaplan–Meier (K-M) curves, in R (https://www.r-project.org/), and MACE incidences in subgroups were compared using the log-rank test. Baseline patient characteristics were compared between patients with DNL and LST/LVST and among DM and non-DM patients.

Continuous variables are presented as the means ± standard errors and categorical data are presented as counts and percentages. Differences between continuous variables were compared using independent *t*-tests, and those between categorical variables were compared using the χ^2^-test or Fisher's exact test to assess the interaction between lesion types and baseline clinical, laboratory index, or angiographic characteristics. We conducted the subgroup analysis by stratification into WBC, other cell counts, D-dimer levels, etc. to access the association between the MACEs and various parameters.

Propensity score matching (PSM) is an increasingly utilized statistical method in non-randomized and observational research in order to make estimation of the influence of the treatment on endpoints accurately (5). It removes the confounding bias factors from the observational cohorts by matching already treated subjects with observational subjects to reduce the unwanted influences of covariates, help account for such imbalances, and allow for proper measurement of the intended variable (6). PSM was employed to adjust for potential confounders and to minimize the impact of selection bias on the comparison between the DM and non-DM groups. We used the one-to-one nearest-neighbor matching for the PSM of patients in different groups, with a caliper width equal to 0.2 of the standard deviation (SD). The procedure yielded 1,178 matched pairs among DNL patients and 108 matched pairs among LST/VLST patients.

The event-free survival rates among groups were calculated by the Kaplan–Meier analysis using method of propensity match and compared by the log-rank test. The confounder factors of models include age, gender, history of hypertension, history of PCI, history of coronary artery bypass graft, history of chronic kidney disease, Killip classification, high-sensitivity C-reactive protein, and estimated glomerular filtration rate. The Mantel–Cox method was used to calculate hazard ratios (HRs) and 95% confidence intervals (CIs) for comparisons of clinical outcomes, including MACEs and all-cause death, between groups, and the log-rank test was used to calculate corresponding *P*-values. We conducted two-sided analyses to allow conventional interpretation of results, and a *P* < 0.05 was considered statistically significant. Missing data were handled by single imputation.

Most of the statistical analyses were conducted using R version I 386 3.6.2 (R Foundation for Statistical Computing, Vienna, Austria). Other analyses were performed using SPSS Statistics version 20.0 (SPSS, Inc., Chicago, IL).

## Results

Table 1 presents the baseline demographic data, indicators of serum inflammation, lipids, angiographic features, and procedural characteristics of the entire study population. In total, 3,941 patients were divided into the DM group (1,178 DNL and 108 LST/VLST lesions) and non-DM group (2,483 DNL and 172 LST/VLST lesions). The mean ages of the patients were 60.51 ± 0.33 and 62.44 ± 0.95 years in the DNL and LST/VLST groups among the DM patients, respectively. Compared with the non-DM patients, subgroups of patients with LST/VLST among the DM patients were older (62.44 ± 0.95 years vs. 59.46 ± 0.80 years, *P* = 0.018), had a smaller proportion of men (70.4% years vs. 82.6%, *P* = 0.013), and underwent PCI (89.8 vs. 80.8%, *P* = 0.003). After PSM, these baseline differences were almost balanced between the two groups.

**Table 1:** Baseline characteristics of entire population.

**Variables**	***De novo*** **lesion patients**			**Propensity-matched** ***de novo*** **lesion patients**			**LST/VLST patients**			**Propensity-matched LST/VLST patients**		
	**DM *De novo* lesion (*N* = 1,178)**	**Non-DM *De novo* lesion (*N* = 2,483)**	**P_**1**_**	**DM *De novo* lesion (*N* = 1,178)**	**Non-DM *De novo* lesion (*N* = 1,178)**	**P_1_^′^**	**DM LST/VLST (*N* = 108)**	**Non-DM LST/VLST (*N* = 172)**	**P2**	**DM LST/VLST (*N* = 108)**	**Non-DM LST/VLST (*N* = 108)**	**P2^**′**^**
Age (years)	60.5 ± 11.3	58.0 ± 12.3	<0.001^*^	60.5 ± 11.3	66.6 ± 11.1	<0.001^*^	62.4 ± 9.8	59.46 ± 8.8	0.018^*^	62.4 ± 9.8	61.5 ± 12.5	0.529
Male [% (*n*)]	861 (73.1%)	2,027 (81.6%)	<0.001^*^	861 (73.1%)	723 (61.4%)	<0.001^*^	76 (70.4%)	142 (82.6%)	0.013^*^	76 (70.4%)	78 (72.2%)	0.764
Heart rate (beats/min)	78.7 ± 15.4	76.9 ± 15.3	0.177	78.7 ± 15.4	77.0 ± 16.0	0.008^*^	77.9 ± 14.8	75.99 ± 1.18	0.859	77.9 ± 14.8	76.5 ± 14.2	0.506
SBP (mmHg)	125.7 ± 17.7	123.5 ± 18.1	0.001^*^	125.7 ± 17.7	124.4 ± 18.8	0.106	127.5 ± 18.7	124.98 ± 1.64	0.979	127.5 ± 18.7	125.5 ± 20.0	0.441
DBP (mmHg)	73.8 ± 12.4	74.4 ± 12.9	0.185	73.8 ± 12.4	72.8 ± 7.4	0.049^*^	74.6 ± 12.2	72.53 ± 1.43	0.630	74.6 ± 12.2	76.4 ± 12.7	0.299
EF at admission	53.4 ± 7.9	54.0 ± 7.3	0.033	53.4 ± 7.9	53.5 ± 7.4	0.712	52.3 ± 8.5	51.40 ± 0.63	0.392	52.3 ± 8.5	50.6 ± 8.3	0.156
**Risk factors**												
Hypertension [% (*n*)]	769 (65.3%)	1,149 (58.4%)	<0.001^*^	769 (65.3%)	762 (64.7%)	0.762	74 (68.5%)	107 (62.2%)	0.282	74 (68.5%)	69 (63.9%)	0.472
Hyperlipidemia [% (*n*)]	997 (94.0%)	2,031 (91.3%)	0.008^*^	997 (94.0%)	940 (89.7%)	<0.001^*^	91 (95.8%)	143 (91.7%)	0.158	91 (95.8%)	87 (88.8%)	0.069
Smoking [% (*n*)]	622 (58.3%)	1,553 (69.3%)	<0.001^*^	622 (58.3%)	574 (54.0%)	0.046	58 (61.1%)	103 (64.8%)	0.321	58 (61.1%)	53 (53.5%)	0.290
Previous PCI [% (*n*)]	122 (10.4%)	164 (6.6%)	<0.001^*^	122 (10.4%)	86 (7.3%)	0.009	97 (89.8%)	139 (80.8%)	0.030^*^	97 (89.8%)	84 (77.8%)	0.016
Previous CABG [% (*n*)]	24 (2.0%)	17 (0.7%)	0.001^*^	24 (2.0%)	9 (0.8%)	0.009	1 (0.9%)	1 (0.6%)	0.624	1 (0.9%)	1 (0.9%)	1.000
CKD [% (*n*)]	107 (9.1%)	170 (6.8%)	0.019^*^	107 (9.1%)	118 (10.0%)	0.441	11 (10.2%)	12 (7.0%)	0.231	11 (10.2%)	11 (10.2%)	1.000
**Comorbidities**												
Malignancy	12 (1.0%)	34 (1.4%)	0.374	12 (1.0%)	27 (2.3%)	0.015	1 (0.9%)	2 (1.2%)	0.851	1 (0.9%)	2 (1.9%)	0.561
Arrhythmology	284 (24.1%)	625 (25.2%)	0.487	284 (24.1%)	359 (30.5%)	<0.001	21 (19.4%)	48 (27.9%)	0.110	21 (19.4%)	36 (33.3%)	0.021
Alimentary ulcer	64 (5.4%)	157 (6.3%)	0.291	64 (5.4%)	80 (6.8%)	0.169	–	–	–	–	–	–
Hypoproteinemia	17 (1.4%)	18 (0.7%)	0.037	17 (1.4%)	14 (1.2%)	0.588	2 (1.9%)	0 (0.0%)	0.073	2 (1.9%)	0 (0.0%)	0.155
Pulmonary disease	81 (6.9%)	147 (5.9%)	0.264	81 (6.9%)	104 (8.8%)	0.078	–	–	–	–	–	–
Gastritis	95 (8.1%)	216 (8.7%)	0.520	95 (8.1%)	115 (9.8%)	0.148	4 (3.7%)	15 (8.7%)	0.104	4 (3.7%)	10 (9.3%)	0.097
Reflux esophagitis	259 (22.0%)	527 (21.2%)	0.600	259 (22.0%)	245 (20.8%)	0.482	0 (0.0%)	1 (0.6%)	1.000	0 (0.0%)	1 (0.9%)	0.316
Cardiomyopathy	9 (0.8%)	8 (0.3%)	0.066	9 (0.8%)	4 (0.3%)	0.164	1 (0.9%)	2 (1.2%)	1.000	1 (0.9%)	2 (1.9%)	0.561
Respiratory failure	13 (1.1%)	12 (0.5%)	0.033	13 (1.1%)	9 (0.8%)	0.392	1 (0.9%)	0 (0.0%)	0.386	1 (0.9%)	0 (0.0%)	0.316
**Laboratory examinations**												
HDL (mg/dl)	1.87 ± 1.37	1.62 ± 0.02	<0.001^*^	1.87 ± 1.37	1.49 ± 0.93	<0.001^*^	1.80 ± 1.31	1.57 ± 0.85	0.082	1.80 ± 1.31	1.58 ± 0.98	0.163
LDL (mg/dl)	2.69 ± 0.90	2.81 ± 0.02	<0.001^*^	2.69 ± 0.90	2.75 ± 0.90	0.158	2.29 ± 0.91	2.45 ± 1.05	0.199	2.29 ± 0.91	2.44 ± 0.95	0.244
Triglycerides (mg/dl)	1.02 ± 0.27	1.06 ± 0.01	0.027^*^	1.02 ± 0.27	1.10 ± 0.29	0.001^*^	1.06 ± 0.40	1.05 ± 0.32	0.816	1.06 ± 0.40	1.09 ± 0.30	0.576
LPA (g/L)	252.87 ± 235	269.58 ± 4.95	0.052	252.87 ± 235.70	284.70 ± 250.69	0.002	264.45 ± 242.23	328.38 ± 312	0.071	264.45 ± 242.23	319.63 ± 298.18	0.137
hs-CRP	7.88 ± 5.01	7.54 ± 0.10	0.054	7.88 ± 5.01	7.90 ± 5.03	0.913	7.27 ± 4.71	6.53 ± 4.75	0.205	7.27 ± 4.71	6.78 ± 4.83	0.451
D-dimer of baseline	0.74 ± 1.83	0.53 ± 0.04	0.533	0.74 ± 1.83	0.77 ± 1.50	0.645	0.96 ± 2.21	0.77 ± 2.11	0.484	0.96 ± 2.21	0.99 ± 2.59	0.937
Peak level of D-dimer	1.14 ± 2.57	0.91 ± 0.05	0.524	1.14 ± 2.57	1.28 ± 2.38	0.247	1.64 ± 0.64	1.13 ± 2.63	0.238	1.64 ± 0.64	1.46 ± 3.22	0.747
Crea	82.69 ± 28.29	81.30 ± 0.45	0.138	82.69 ± 28.29	81.39 ± 26.30	0.249	82.53 ± 25.90	81.93 ± 22.21	0.836	82.53 ± 25.90	82.76 ± 24.65	0.946
eGFR (MDRD)	87.89 ± 65.59	91.78 ± 1.83	0.191	87.89 ± 65.59	83.70 ± 64.67	0.119	81.90 ± 22.42	91.51 ± 98.04	0.318	81.90 ± 22.42	82.27 ± 22.22	0.904
Peak level of TnI	8.44 ± 16.94	4.09 ± 0.28	0.177	8.44 ± 16.94	9.10 ± 15.98	0.585	6.37 ± 4.31	16.44 ± 26.99	0.078	6.37 ± 4.31	17.78 ± 26.90	0.048
Glycemia	3.53 ± 2.72	3.02 ± 1.62	<0.001^*^	3.53 ± 2.72	3.06 ± 1.66	<0.001^*^	4.05 ± 3.34	3.05 ± 1.63	0.002	4.05 ± 3.34	3.11 ± 1.76	0.015
**Discharge medication regimen**												
Statin [% (*n*)]	1,112 (94.4%)	2,316 (93.3%)	0.218	1,112 (94.4%)	1,122 (95.2%)	0.353	102 (94.4%)	159 (92.4%)	0.349	102 (94.4%)	100 (92.6%)	0.580
Aspirin [% (*n*)]	1,160 (98.5%)	2,467 (99.4%)	0.015^*^	1,160 (98.5%)	1,168 (99.2%)	0.128	106 (98.1%)	170 (98.8%)	0.500	106 (98.1%)	106 (98.1%)	1.000
Clopidogrel [% (*n*)]	924 (78.4%)	1,912 (77.0%)	0.352	924 (78.4%)	920 (78.1%)	0.842	75 (69.4%)	121 (70.3%)	0.488	75 (69.4%)	81 (75.0%)	0.362
Ticagrelor [% (*n*)]	244 (20.9%)	553 (22.4%)	0.303	244 (20.9%)	553 (22.4%)	0.290	31 (29.2%)	50 (29.1%)	0.540	31 (29.2%)	27 (25.0%)	0.485
ACEI [% (*n*)]	721 (61.2%)	1,547 (62.3%)	0.536	721 (61.2%)	709 (60.2%)	0.613	61 (56.5%)	105 (61.0%)	0.263	61 (56.5%)	64 (59.3%)	0.679
ARB [% (*n*)]	124 (10.5%)	195 (7.9%)	0.008^*^	124 (10.5%)	97 (8.2%)	0.056	13 (12.0%)	18 (10.5%)	0.412	13 (12.0%)	12 (11.1%)	0.832
ACEI/ARB [% (*n*)]	843 (71.6%)	1,741 (70.1%)	0.393	843 (71.6%)	805 (68.3%)	0.088	74 (68.5%)	123 (71.5%)	0.344	74 (68.5%)	76 (70.4%)	0.768
Beta-Blockers [% (*n*)]	1,040 (88.3%)	2,150 (86.6%)	0.154	1,040 (88.3%)	1,011 (85.8%)	0.075	89 (82.4%)	161 (93.6%)	0.003^*^	89 (82.4%)	98 (90.7%)	0.072
Diuretic [% (*n*)]	365 (31.0%)	674 (27.1%)	0.017^*^	365 (31.0%)	366 (31.1%)	0.964	35 (32.4%)	66 (38.4%)	0.189	35 (32.4%)	46 (42.6%)	0.122
Spironolactone [% (*n*)]	246 (20.9%)	530 (21.3%)	0.762	246 (20.9%)	267 (22.7%)	0.295	26 (24.1%)	56 (32.6%)	0.083	26 (24.1%)	39 (36.1%)	0.054
P2Y12 inhibitors	1,167 (99.1%)	2,465 (99.3%)	0.550	1,167 (99.1%)	1,166 (99.0%)	0.834	106 (98.1%)	171 (99.4%)	0.331	106 (98.1%)	108 (100.0%)	0.155
**Lesion and procedural characteristics**												
Lesion length, mm	28.53 ± 16.33	27.01 ± 15.16	<0.001^*^	28.53 ± 16.33	27.99 ± 16.44	0.366	26.01 ± 15.80	27.72 ± 15.31	0.369	26.01 ± 15.80	27.2 ± 16.0	0.596
Lesion diameter, mm	3.05 ± 0.52	3.12 ± 0.51	0.006	3.05 ± 0.52	3.08 ± 0.53	0.055	3.03 ± 0.42	3.06 ± 0.49	0.657	3.03 ± 0.42	3.0 ± 0.4	0.881
Degree of lesion stenosis	97.00 ± 0.16	97.22 ± 0.11	0.249	97.02 ± 5.28	97.16 ± 5.97	0.425	98.34 ± 4.19	98.40 ± 4.15	0.431	98.34 ± 4.19	98.86 ± 2.89	0.93
Bifurcation lesion	405 (34.4%)	862 (34.7%)	0.853	405 (34.4%)	413 (35.1%)	0.729	27 (25.0%)	49 (28.5%)	0.310	27 (25.0%)		0.178
TIMI after PCI			0.112			0.585			0.748			0.441
0	25 (2.1%)	28 (1.1%)	–	25 (2.1%)	17 (1.4%)	–	5 (4.6%)	5 (2.9%)	-	5 (4.6%)	2 (1.9%)	
1	5 (0.4%)	9 (0.4%)	–	5 (0.4%)	6 (0.5%)	–	0 (0.0%)	1 (0.6%)	-	0 (0.0%)	0 (0.0%)	
2	23 (2.0%)	42 (1.7%)	–	23 (2.0%)	27 (2.3%)	–	1 (0.9%)	2 (1.2%)	-	1 (0.9%)	2 (1.9%)	
3	1,125 (95.5%)	2,404 (96.8%)	–	1,125 (95.5%)	1,128 (95.8%)	–	102 (94.4%)	164 (95.3%)	-	102 (94.4%)	104 (96.3%)	
PTCA	1,031 (87.5%)	2,176 (87.6%)	0.915	1,031 (87.5%)	1,047 (88.9%)	0.307	100 (92.6%)	158 (91.9%)	0.509	100 (92.6%)	103 (95.4%)	0.391
Thrombus aspiration	456 (38.7%)	1,085 (43.7%)	0.005^*^	456 (38.7%)	489 (41.5%)	0.165	39 (36.1%)	76 (44.2%)	0.113	39 (36.1%)	45 (41.7%)	0.402
Stent implantation	1,043 (88.5%)	2,264 (91.2%)	0.014^*^	1,043 (88.5%)	1,063 (90.2%)	0.181	61 (56.5%)	118 (68.6%)	0.027^*^	61 (56.5%)	75 (69.4%)	0.049
IABP	120 (10.2%)	230 (9.3%)	0.400	120 (10.2%)	136 (11.5%)	0.290	11 (10.2%)	21 (12.2%)	0.377	11 (10.2%)	11 (10.2%)	1.000
Multi-vessel disease	958 (81.3%)	1,780 (71.7%)	<0.001^*^	958 (81.3%)	903 (76.7%)	0.005	86 (79.6%)	130 (75.58%)	0.432	86 (79.6%)	79 (73.1%)	0.262
Type of ACC			0.087			0.010			0.580			0.590
A	25 (2.1%)	42 (1.7%)	–	25 (2.1%)	13 (1.1%)	–	2 (1.9%)	4 (2.33%)	–	2 (1.9%)	3 (2.8%)	–
B1	90 (7.6%)	147 (5.9%)	–	90 (7.6%)	59 (5.0%)	–	5 (4.6%)	7 (4.07%)	–	5 (4.6%)	4 (3.7%)	–
B2	132 (11.2%)	323 (13.0%)	–	132 (11.2%)	144 (12.2%)	–	11 (10.2%)	10 (5.81%)	–	11 (10.2%)	6 (5.6%)	–
C	931 (79.0%)	1,971 (79.4%)	–	931 (79.0%)	962 (81.7%)	–	90 (83.3%)	151 (87.79%)	–	90 (83.3%)	95 (88.0%)	–
Site of lesion			0.050			0.092			0.097			0.006
LAD	170 (14.4%)	385 (15.5%)	–	170 (14.4%)	148 (12.6%)	–	23 (21.3%)	20 (11.6%)	–	23 (21.3%)	7 (6.5%)	–
RCA	476 (40.4%)	940 (37.9%)	–	476 (40.4%)	474 (40.2%)	–	35 (32.4%)	49 (28.5%)	–	35 (32.4%)	31 (28.7%)	–
LAD	493 (41.9%)	1,091 (43.9%)	–	493 (41.9%)	511 (43.4%)	–	47 (43.5%)	99 (57.6%)	–	47 (43.5%)	68 (63.0%)	–
LM	29 (2.5%)	61 (2.5%)	–	29 (2.5%)	42 (3.6%)	–	3 (2.8%)	3 (1.7%)	–	3 (2.8%)	1 (0.9%)	–
Vein graft	10 (0.8%)	6 (0.2%)	–	10 (0.8%)	3 (0.3%)	–	0 (0.0%)	1 (0.6%)	–	0 (0.0%)	1 (0.9%)	–
**Endpoint events**												
MACE	141 (12.0%)	209 (8.4%)	0.001^*^	141 (12.0%)	138 (11.7%)	0.848	15 (13.9%)	30 (17.4%)	0.269	15 (13.9%)	22 (20.4%)	0.206
All caused mortality	82 (7.0%)	106 (4.3%)	0.001^*^	82 (7.0%)	85 (7.2%)	0.810	6 (5.6%)	17 (9.9%)	0.144	6 (5.6%)	13 (12.0%)	0.093
Cardiovascular death	50 (4.2%)	71 (2.9%)	0.037^*^	50 (4.2%)	56 (4.8%)	0.551	5 (4.6%)	11 (6.4%)	0.386	5 (4.6%)	9 (8.3%)	0.269
Recurrence MI	43 (3.7%)	70 (2.8%)	0.184	43 (3.7%)	70 (2.8%)	0.176	6 (5.6%)	10 (5.8%)	0.576	6 (5.6%)	7 (6.5%)	0.775
Ischemic stroke	21 (1.8%)	42 (1.7%)	0.892	21 (1.8%)	42 (1.7%)	0.845	5 (4.6%)	3 (1.7%)	0.149	5 (4.6%)	2 (1.9%)	0.249

*DM, diabetes mellitus; LST, late stent thrombosis; VLST, very late stent thrombosis; SBP, systolic blood pressure; DBP, diabetes blood pressure; EF, ejection fraction; PCI, percutaneous coronary intervention; CABG, coronary artery bypass graft; AF, atrial fibrillation; CKD, chronic kidney disease; HDL, high-density lipoprotein; LDL, low-density lipoprotein; LPA, lipase activator; hs-CRP, high-sensitivity C-reactive protein; eGFR, estimated glomerular filtration rate; ACEI, angiotensin-converting enzyme inhibitor; ARB, angiotensin receptor blocker; TnI, troponin; TIMI, thrombolysis in myocardial infarction; PTCA, percutaneous transluminal coronary angioplasty; IABP, intra-aortic balloon pump; MACE, major adverse cardiovascular event; MI, myocardial infarction*.

* *P < 0.05*.

**Figure 2:**
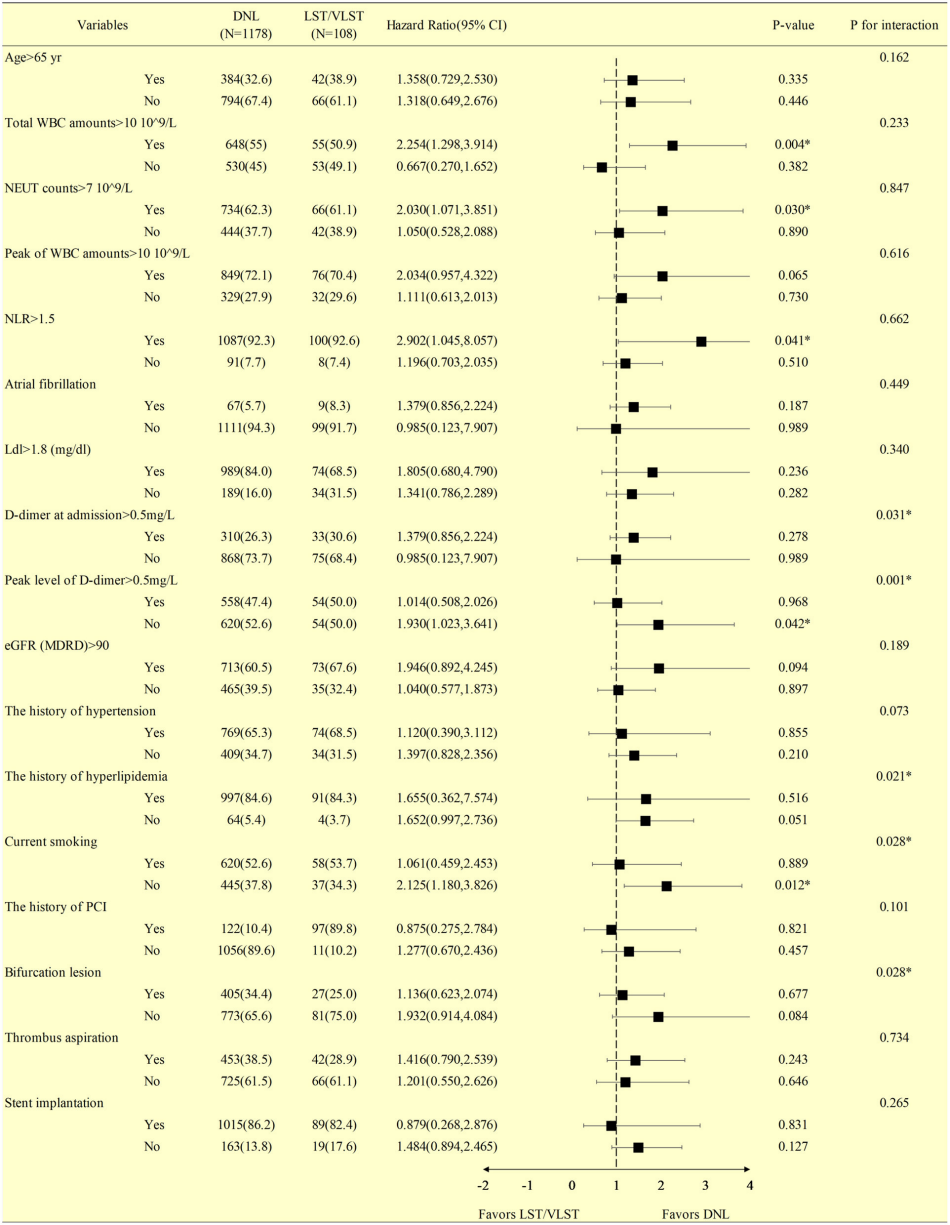
Stratified analysis of the revascularization at a median follow-up of 698 days in patients with DNL or LST/VLST lesion. Values are *n* (%). The primary endpoint is revascularization. *Interaction is for risk ratio −2 to 1 year and risk ratio 1–4 years for LST/VLST and DNL. DNL, *de novo* lesion; LST, late stent thrombosis; VLST, very late stent thrombosis; CI, confidence interval; WBC, white blood cell; NEUT, neutral granular cell counts; NLR, neutrophil–lymphocyte ratio; LDL, low-density lipoprotein; eGFR, estimated glomerular filtration rate; PCI, percutaneous coronary intervention.

The findings of revascularization were consistent across the stratified subgroup analyses, including variables representing serum inflammation, lipids, and thrombus levels (Figure 2). In particular, in the subgroup with a total white blood cell (WBC) count >10 × 10^9^/L (*P* = 0.004, *P*_interaction_ = 0.233), a neutral granular cell count >7 × 10^9^/L (*P* = 0.030, *P*_interaction_ = 0.847), neutrophil–lymphocyte ratio (NLR) >1.5 (*P* = 0.041, *P*_interaction_ = 0.662), peak D-dimer level <0.5 mg/L (*P* = 0.042, *P*_interaction_ = 0.001), and not currently smoking (*P* = 0.012, *P*_interaction_ = 0.028), DNLs were more likely to be revascularized than LST/VLST lesions at a median follow-up of 698 days.

Table 2 describes the associations of MACEs, stratified according to the immunity index, and lipid and inflammatory marker levels during treatment. Among the DM patients with DNLs, each unit increase in age was associated with a 53.6% increase in the risk of MACEs (HR: 1.536, 95% CI, 1.300–1.815, *P* < 0.0001). Older age (≥65 years) was associated with a significantly greater risk of MACEs (HR, 2.115; 95% CI, 1.519–2.944; *P* < 0.0001). Furthermore, each SD increase in the peak WBC level was associated with a 50.1% increase in the risk of MACEs (HR, 1.501; 95% CI, 1.208–1.864; *P* = 0.0002). When stratifying the DM population with DNLs according to the D-dimer levels (baseline and peak level) <0.5 vs. ≥0.5 mg/L, the group with the higher D-dimer level at baseline had a 2.066-fold higher risk of MACEs (*P* < 0.0001). Patients with a higher peak D-dimer level had a 1.877-fold higher risk of MACEs (*P* = 0.001) than those with lower levels. Among the DM patients with LST/VLST lesions, each unit increase in age was associated with a 75.9% increase in the risk of MACEs (HR: 1.759, 95% CI, 1.052–2.940, *P* = 0.032). Furthermore, each SD increase in the peak D-dimer level was associated with a 59.7% increase in the risk of MACEs (HR, 1.597; 95% CI, 1.110–2.295; *P* = 0.041). Each SD increase in the level of lipoprotein A was associated with a 58.1% decrease in the risk of MACEs (HR, 0.419; 95% CI, 0.179–0.979; *P* = 0.045).

**Table 2:** Association between MACE and different subgroups of variables among patients with DM.

**Subgroups**	**DNL patients with DM**		**LST/VLST patients with DM**	
	**HR (95% CI)**	***P*-value**	**HR (95% CI)**	***P*-value**
**Age**				
Age per SD	1.536 (1.300, 1.815)	<0.0001^*^	1.759 (1.052, 2.940)	0.032^*^
<65 yr	1 [reference]		1 [reference]	
≥65 yr	2.115 (1.519, 2.944)	<0.0001^*^	2.217 (0.789, 6.232)	0.131
**Total WBC amounts**				
Total WBC per SD	1.197 (0.936, 1.530)	0.153	1.202 (0.727, 1.988)	0.474
<10 × 10^9^/L	1 [reference]		1 [reference]	
≥10 × 10^9^/L	1.306 (0.761, 2.241)	0.333	0.897 (0.240, 3.348)	0.871
**Peak of WBC amounts**				
Peak of WBC per SD	1.501 (1.208, 1.864)	0.0002^*^	1.023 (0.978, 1.176)	0.752
<10 × 10^9^/L	1 [reference]		1 [reference]	
≥10 × 10^9^/L	1.128 (0.575, 2.211)	0.726	0.867 (0.202, 3.722)	0.848
**NLR**				
NLR per SD	1.289 (1.035, 1.603)	0.023^*^	0.920 (0.463, 1.828)	0.812
<1.5	1 [reference]		1 [reference]	
≥1.5	1.128 (0.575, 2.211)	0.726	0.419 (0.050, 3.517)	0.423
**Hs-CRP**				
Hs-CRP per SD	1.021 (0.864, 1.207)	0.806	0.912 (0.533, 1.561)	0.738
< Median	1 [reference]		1 [reference]	
≥Median	1.040 (0.744, 1.453)	0.819	0.511 (0.179, 1.454)	0.208
**Lpa**				
Lpa per SD	0.908 (0.714, 1.153)	0.428	0.419 (0.179, 0.979)	0.045^*^
< Median	1 [reference]		1 [reference]	
≥Median	1.092 (0.784, 1.521)	0.602	0.312 (0.099, 0.986)	0.047^*^
**TG**				
TG per SD	0.952 (0.806, 1.125)	0.056	1.292 (0.917, 1.819)	0.143
< Median	1 [reference]		1 [reference]	
≥Median	1.108 (0.795, 1.546)	0.544	1.123 (0.396, 3.183)	0.827
**Ldl**				
Ldl per SD	1.046 (0.880, 1.243)	0.609	0.661 (0.343, 1.275)	0.217
<1.8 (mg/dl)	1 [reference]		1 [reference]	
≥1.8 (mg/dl)	1.176 (0.708, 1.923)	0.532	0.647 (0.233, 1.802)	0.405
**D-dimer at admission**				
D-dimer per SD	1.150 (1.050, 1.260)	0.0026^*^	1.139 (0.828, 1.566)	0.425
<0.5	1 [reference]		1 [reference]	
≥0.5	2.066 (1.448, 2.948)	<0.0001^*^	2.089 (0.635, 6.871)	0.225
**Peak value of D-dimer**				
D-dimer per SD	1.103 (0.993, 1.226)	0.067	1.597 (1.11, 2.295)	0.041^*^
<0.5	1 [reference]		1 [reference]	
≥0.5	1.877 (1.291, 2.728)	0.001^*^	2.742 (0.646, 11.635)	0.172
**eGFR (MDRD)**				
eGFR per SD	0.906 (0.732, 1.122)	0.365	0.681 (0.421, 1.103)	0.118
<90	1 [reference]		1 [reference]	
≥90	0.681 (0.473, 0.980)	0.359^*^	0.314 (0.071, 1.394)	0.128

*SD, standard deviation; HR, hazard ratio; CI, confidence interval; DNL, de novo lesion; LST, late stent thrombosis; VLST, very late stent thrombosis; DM, diabetes mellitus; MACE, major adverse cardiovascular events; yr, year; WBC, white blood cell; NLR, neutrophil–lymphocyte ratio; LDL, low-density lipoprotein; LPA, lipase activator; hs-CRP, high-sensitivity C-reactive protein; eGFR, estimated glomerular filtration rate;*

* *P < 0.05*.

Multivariate Cox regression of the DNL and LST/VLST patients with DM is summarized in Table 3. Age >65 years was the only independent predictor for the composite endpoint among the DM patients with DNL, and no predictor of the composite endpoint was identified in this subgroup.

**Table 3:** Multivariate Cox regression analysis of patients with divided into DNL and LST/VLST.

**Variables**	**DNL patients with DM (*****N*** **=** **1,177)**					**LST/VLST patients with DM (*****N*** **=** **108)**				
	**Coefficient**	**HR [exp(coef)]**	**95% CI upper**	**95% CI lower**	***P*-value**	**Coefficient**	**HR [exp(coef)]**	**95% CI upper**	**95% CI lower**	***P*-value**
Male	−0.2879	0.7498	0.4899	1.1476	0.1848	1.4519	4.2712	0.3730	48.9158	0.2432
Age >65 yr	0.4245	1.5289	1.0016	2.3338	0.0492^*^	0.0897	1.0938	0.1260	9.4931	0.9352
History of hypertension	0.2176	1.2431	0.8039	1.9223	0.3279	0.8767	2.4029	0.2104	27.4361	0.4804
History of hyperlipidemia	−0.0493	0.9519	0.4572	1.9820	0.8952	−0.1430	0.8667	0.0288	26.1243	0.9344
LDL-C ≥median	0.0417	1.0426	0.5927	1.8340	0.8850	−0.2628	0.7689	0.1151	5.1358	0.7862
Hs-CRP ≥median	−0.2108	0.8100	0.5456	1.2025	0.2958	−1.2242	0.2940	0.0320	2.7025	0.2794
eGFR (MDRD) ≥90	−0.0398	0.9610	0.6163	1.4984	0.8605	−2.1170	0.1204	0.0079	1.8250	0.1269
D-dimer at admission ≥0.5	0.2899	1.3363	0.7108	2.5123	0.3681	18.3358	Inf.	0.0000	Inf.	0.9985
Peak value of D-dimer ≥0.5	0.2484	1.2819	0.6880	2.3884	0.4341	−16.6900	Inf.	0.0000	Inf.	0.9986
Lpa ≥median	0.2343	1.2640	0.8548	1.8693	0.2405	−1.6927	0.1840	0.0254	1.3350	0.0941
TG ≥median	0.0968	1.1017	0.7478	1.6229	0.6242	−0.1185	0.8882	0.1324	5.9576	0.9029

*DNL, de novo lesion; LST, late stent thrombosis; VLST, very late stent thrombosis; DM, diabetes mellitus; yr, year; TG, triglyceride; LDL, low-density lipoprotein; LPA, lipase activator; hs-CRP, high-sensitivity C-reactive protein; eGFR, estimated glomerular filtration rate; MDRD, modification of diet in renal disease equation; HR indicates hazard ratio; CI, confidence interval;*

* *P < 0.05*.

**Figure 3:**
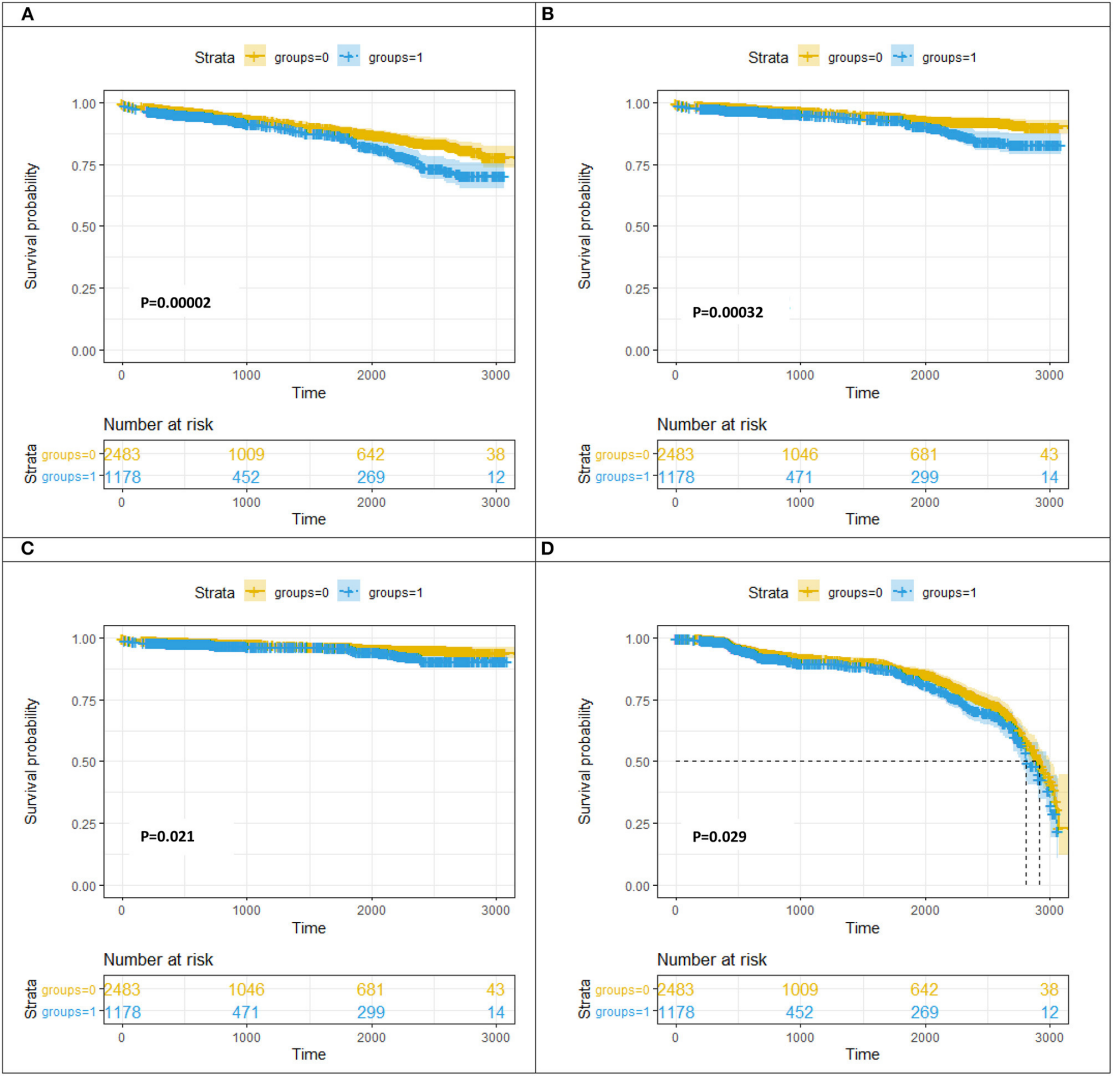
Kaplan–Meier curve analysis for MACE **(A)**/all-cause death **(B)**/cardiac-caused death **(C)**/revascularization **(D)** at follow-up between the DM group and the non-DM group. DNL, *de novo* lesion; LST, late stent thrombosis; VLST, very late stent thrombosis groups = 0, non-diabetes mellitus group; groups = 1, diabetes mellitus group.

Table 4 and Figure 3 present the cumulative incidence of clinical outcomes by KM analysis at 2 years after PCI. Among the patients with DNL, the cumulative incidences of MACEs (8.42 vs. 11.97%, log rank = 0.0002), all-cause death (4.27 vs. 6.91%, log rank = 0.00032), cardiac-related death (2.86 vs. 4.24%, log rank = 0.021), and revascularization (1.34 vs. 1.49%, log rank = 0.029) were lower in the non-DM group than in the DM group.

**Table 4:** The cumulative incidence of clinical outcomes by Kaplan–Meier analysis at median 698 follow-up days among all enrolled patients.

**Endpoints**	**DNL** ***N*** **=** **2,661**			**LST/VLST** ***N*** **=** **280**		
	**DM (*N* = 1,178)**	**Non-DM (*N* = 2,483)**	**Log rank**	**DM (*N* = 108)**	**Non-DM (*N* = 172)**	**Log rank**
MACE	141 (11.97%)	209 (8.42%)	0.0002^*^	15 (12.89%)	30 (17.44%)	0.94
All-cause death	82 (6.91%)	106 (4.27%)	0.00032^*^	6 (5.56%)	17 (9.88%)	0.44
Cardiac-caused death	50 (4.24%)	71 (2.86%)	0.021^*^	5 (4.63%)	11 (4.40%)	0.87
Recurrence MI	43 (3.65%)	70 (2.82%)	0.12	6 (5.56%)	10 (5.81%)	0.79
Revascularization	175 (1.49%)	332 (1.34%)	0.029^*^	21 (19.44%)	27 (15.70%)	0.085
Ischemic stroke	21 (1.78%)	42 (1.69%)	0.67	5 (4.63%)	3 (1.74%)	0.088

*LST, late stent thrombosis; VLST, very late stent thrombosis; DM, diabetes mellitus; MACE, major adverse cardiovascular events; MI, myocardial infarction*.

* *P < 0.05*.

Table 5 presents the cumulative incidence of clinical outcomes by KM analysis at a median follow-up of 698 days after PSM. Among the patients with DNL, the cumulative incidences of MACEs (crude HR, 1.497, 95% CI, 1.209–1.853, log rank *P* < 0.001), all-cause death (crude HR, 1.687, 95% CI, 1.264–2.251, log rank *P* < 0.001), and cardiac-related death (crude HR, 1.529, 95% CI, 1.065–2.196, log rank *P* = 0.021) were lower in the non-DM group than in the DM group. After adjusting for age, sex, history of hypertension, history of PCI, history of CABG, history of CKD, Killip classification, high-sensitivity C-reactive protein, and estimated glomerular filtration rate, the cumulative incidences of MACEs (adjusted HR, 1.269, 95% CI, 1.023–1.576, log rank *P* = 0.031) and all-cause death (crude HR, 1.355, 95% CI, 1.011–1.815, log rank *P* = 0.042) were lower in the non-DM group than in the DM group after PSM.

**Table 5:** Primary outcomes by propensity score matching before and after fully adjustment.

**Endpoints**	**Propensity-matched DNL patients**				**Propensity-matched LST/VLST**			
	**DM (*****N*** **=** **1,178) vs. non-DM (*****N*** **=** **1,178)**				**DM (*****N*** **=** **108) vs. non-DM (*****N*** **=** **108)**			
	**Crude HR (95% CI)**	***P*-value**	**Adjusted HR (95% CI)**	**Adjusted *P*-value**	**Crude HR (95% CI)**	***P*-value**	**Adjusted HR (95% CI)**	**Adjusted *P*-value**
MACE	1.497 (1.209, 1.853)	<0.001^*^	1.269 (1.023, 1.576)	0.031^*^	0.858 (0.442, 1.668)	0.652	0.629 (0.290, 1.363)	0.240
All-cause death	1.687 (1.264, 2.251)	<0.001^*^	1.355 (1.011, 1.815)	0.042^*^	0.578 (0.217, 1.544)	0.274	0.201 (0.049, 0.829)	0.027^*^
Cardiac-caused death	1.529 (1.065, 2.196)	0.021^*^	0.861 (0.588, 1.261)	0.442	0.723 (0.236, 2.217)	0.571	0.121 (0.018, 0.836)	0.032^*^
Recurrence MI	1.348 (0.922, 1.971)	0.123	2.398 (0.539, 10.675)	0.251	1.025 (0.344, 3.054)	0.965	2.335 (0.593, 9.198)	0.225
Revascularization	1.009 (0.828, 1.230)	0.929	1.018 (0.832, 1.247)	0.860	0.462 (0.210, 1.018)	0.055	0.503 (0.187, 1.352)	0.173
Ischemic stroke	1.120 (0.663, 1.892)	0.671	1.006 (0.592, 1.709)	0.982	3.233 (0.627, 16.677)	0.161	2.290 (0.429, 12.214)	0.332

*HR indicates hazard ratio; DNL, de novo lesion; LST, late stent thrombosis; VLST, very late stent thrombosis; DM, diabetes mellitus; MACE, major adverse cardiovascular events; MI, myocardial infarction*.

* *P < 0.05. Adjusted P values are HRs (95% confidence intervals) from models adjusted for age, gender, history of hypertension, history of percutaneous coronary intervention, history of coronary artery bypass graft, history of chronic kidney disease, Killip classification, high-sensitivity-C reaction protein, and estimated glomerular filtration rate*.

The results of the time-to-event analysis for the primary endpoint of MACEs, all-cause death, cardiac-related death, recurrent MI, revascularization, and ischemic stroke at follow-up between the DNL and LST/VLST groups among the DM patients are summarized in Table 6.

**Table 6:** The cumulative incidence of clinical outcomes by Kaplan–Meier analysis at 698 median follow-up days among DM subjects.

**Endpoints**	**DNL *N* = 1,178**	**LST/VLST *N* = 108**	**Log rank**
MACE	141 (11.97%)	15 (12.89%)	0.250
All-cause death	82 (6.91%)	6 (5.56%)	0.736
Cardiac-caused death	50 (4.24%)	5 (4.63%)	0.720
Recurrence MI	43 (3.65%)	6 (5.56%)	0.320
Revascularization	175 (1.49%)	21 (19.44%)	0.0002^*^
Ischemic stroke	21 (1.78%)	5 (4.63%)	0.033^*^

*LST, late stent thrombosis; VLST, very late stent thrombosis; DM, diabetes mellitus; MACE, major adverse cardiovascular events; MI, myocardial infarction*.

* *P < 0.05*.

## Discussion

### Main Findings

In this study, which involved 3,941 real-world consecutive patients who had undergone primary PCI in China, we obtained the following major findings. The cumulative incidences of MACEs, all-cause death, cardiac-related death, and revascularizations were lower among patients with DNL in the non-DM group than in those in the DM group. Age, WBC count, and baseline and peak D-dimer levels were associated with increased MACEs. In particular, after successful primary PCI, the measurement of baseline and peak D-dimer levels in patients may help identify individuals at higher cardiovascular risk. Additionally, our finding suggests a potential benefit of lowering D-dimer levels among DM patients with DNL. Age and peak D-dimer levels may facilitate the risk stratification of DM patients with LST/VLST.

### Impact of Inflammatory Cells on DNLs and LST/VLST Lesions Among DM Patients

Previous studies showed that an elevated WBC count was correlated with myocardial perfusion disorders (7) and an increased death risk during the first 6 months following MI among patients with ACS (8). Furthermore, a WBC count >10,000/L indicated an increased mortality risk among acute MI patients (9). It has been reported that increased leukocyte counts on admission are associated with congestive heart failure, shock, and the development of worse microvascular injury in patients with ACS (10).

The main mechanism of post-angioplasty restenosis is the binding of leukocyte P-selectin glycoprotein ligand-1 (PSGL-1) to platelet P-selectin, which causes marked neointimal proliferation and thrombo-inflammation, leading to luminal loss (11). Consequently, in our study, we observed that each SD increase in the level of peak WBC counts was associated with a 50.1% increase in the risk of MACEs (HR, 1.501; 95% CI, 1.208–1.864; *P* = 0.0002) among DM patients with DNLs.

The NLR, one of the best-assessed hematological biomarkers, is measured by dividing the neutrophil count by the lymphocyte count; it provides diagnostic and prognostic information in ACS (12). In patients with ST-segment elevation MI (STEMI), a high pre-procedural NLR (>4.9) enables a clinician to predict in-hospital mortality with 70% accuracy and 65% specificity and is associated with both LST and VLST (13). Among patients undergoing angiography or cardiac revascularization, NLR is related to the progression of coronary atherosclerosis (14) and plays the role of a predictor of cardiovascular events and all-cause mortality (15). In the present study, we observed that each SD increase in the peak NLR level was associated with a 28.9% increase in the risk of MACEs (HR, 1.289; 95% CI, 1.035–1.603; *P* = 0.023), which was consistent with a previous report (16) on type 2 DM patients with DNL undergoing primary PCI. However, no significant difference was observed in the subgroup of patients with LST/VLST lesions. Furthermore, Soehnlein et al. (17) examined the contribution of neutrophils to the process of arterial healing after injury and found that neutrophil-derived cathelicidin limited neointima formation and promoted re-endothelialization after stent implantation. However, the potential causal mechanisms are currently still obscure and require further investigation.

### Impact of D-Dimer on DNL and LST/VLST Lesions Among DM Patients

D-dimer, a marker of hypercoagulability, fibrin formation, and thrombin generation, has been implicated in angiogenesis and metastatic spread (18). Increased D-dimer levels were reported to be an independent risk factor for in-hospital MACEs and a long-term risk of cardiovascular disease-related mortality in STEMI patients undergoing primary PCI (19, 20). Over the 6 years of the trial, the D-dimer level was one of several biomarkers that predicted cardiovascular disease events, and the associations remained strong more than 10 years after the initial D-dimer reading (21). The long-term risk indicated by D-dimer levels is related to many pathogenic pathways, including inflammatory and atherosclerotic processes. However, the role of D-dimer levels in predicting outcomes ≥5 years in LST/VLST patients with type 2 DM who have undergone primary PCI has not been as clearly defined. In the present study, DM patients with DNL with high D-dimer levels at baseline had a 2.066-fold increase in the risk of MACEs (*P* < 0.0001) and those with a high peak D-dimer level had a 1.877-fold increase in the risk of MACEs (*P* = 0.001), compared with those with low D-dimer levels. Furthermore, each SD increase in the peak D-dimer value was associated with a 59.7% increase in the risk of MACEs (HR, 1.597; *P* = 0.041) in DM patients with LST/VLST.

### DM and DNL

This study showed that DM patients have significantly higher incidences of MACEs (11.97 vs. 8.42%, *P* = 0.0002), all-cause death (6.91 vs. 4.27%, *P* = 0.00032), cardiac-related death (4.24 vs. 2.86%, *P* = 0.021), and revascularization (1.49 vs. 1.34%, *P* = 0.029) than non-DM patients among patients with DNL. Owing to microvascular dysfunction, thrombus burden, unstable plaque, and diffuse distribution of atherosclerotic lesions, DM patients with ACS generally had higher incidences of LST/VLST (22, 23). Inflammation and accumulation of reactive oxygen species and metabolic cytokines are primary mechanisms of vascular remodeling and progression of adverse myocardial diseases resulting from glycemic variability and hyperglycemia (24–28). Furthermore, a previous study has reported that insulin resistance is higher in patients with cardiovascular disease (29). Various biomarkers are proposed to play a role in the stratification of ACS. Cyr61, which predicts primary endpoints in patients with ACS, is involved in cell adhesion, proliferation, migration, and inflammation (30). Indeed, glycemic variability has previously been shown as an outcome predictor of patients with ACS undergoing PCI (31). Furthermore, the risk for repeat revascularization has been related to DM severity, with insulin-dependent DM having the highest risk factor for repeat revascularization (32). Elevated glucose level is markedly related to sympathetic stimulation, and catecholamine can stimulate glucose release and control hyperglycemia (33). Mechanistically, an increase in the incidence of MACE, mortality, and stroke among DM patients might be a result of direct glucotoxic effects, which lead to the attenuation of endothelium-dependent vasodilatation and myocardial perfusion damage (34). Furthermore, hyperglycemia can cause conformational changes in platelet glycoproteins and affect platelet function and intraplatelet signaling pathways; as a result, more solid coronary clots are formed (35, 36).

### DM and LST/VLST

In the implantation of first-generation DES, the incidence of LST/VLST was correlated with incomplete stent apposition and delayed endothelial coverage, thereby leading to chronic inflammation (37). However, second-generation DES, which is characterized with durable, biodegradable, and biocompatible polymers, is not resistant to LST/VLST (38). The mechanisms of thrombosed stent segments are fibrin deposition and chronic inflammation leading to strut malapposition, delayed healing, and heart remodeling, which are distinct from early ST (37, 39). Previous studies (40, 41) have identified DM as an important clinically independent predictor of poor outcome in ST in the real world of mixed use of bare-metal stents (BMS) and DES. Longer lesion length, smaller vessel size, a higher rate of residual dissections, increasing thrombus burden, and bifurcation lesions might be the underlying reasons for a predisposition of DM patients to ST (42, 43). This study highlights that total WBC count (*P* = 0.021) and neutral granular cell count (*P* = 0.018) were independent risk factors of LST/VLST among DM patients. This is consistent with the severe inflammation status of DM patients with LST/VLST. In addition, this study found a significant increase in the incidence of ischemic stroke in patients with LST/VLST compared with those with DNL (log rank = 0.033). A previous study has reported that neovascularization, fibrin accumulation, and thrombus burden are accompanied by inflammation, which correlated with the early healing of thrombus (44). Occasional accumulation of macrophages, giant cells, and lymphocytes is a main characteristic of the inflammatory response after percutaneous coronary stenting (45). Presence of peristrut eosinophilic material in the plasma might be a marker of endothelial cell leakage. Therefore, it is necessary to compare the effects of hypercholesterolemia using a healthy model.

### DM and In-stent Thrombosis

DM patients have a 2- to 4-fold higher risk of developing in-stent restenosis (ISR) after PCI than non-DM patients and thus deserve additional attention. Although new-generation DES have greatly decreased neointimal proliferation, ISR and late stent failure are common complications and crucial after coronary stenting. A recent study (46) confirmed that a higher hemoglobin A1c (HbA1c) variability in type 2 DM patients was more likely to cause higher incidences of neointimal hyperplasia and ISR and hypothesized that post-prandial glucose variability might be more important than fasting glucose in the development of ISR. Compelling evidence from a notable study (47) has confirmed a significantly increased rate of ISR in DM patients undergoing PCI irrespective of specific treatment modalities, such as BMS, DES, and balloon angioplasty. Another study reported that endothelial dysfunction and impaired bioavailability of endothelium-derived nitric oxide play a critical role in the pathogenesis of post-PCI restenosis (48). The possible mechanisms of glycemic and HbA1c variabilities that affect the progression of ISR in DM patients remain unclear. Previous studies concluded that hyperglycemia (24, 25), insulin resistance (26), and glycemic variability (27) result in adverse vascular and myocardial remodeling directly and indirectly by stimulating the production of inflammatory factors, metabolic cytokines, and reactive oxygen species. This is consistent with our finding that the prognosis of DNL outperformed ISR, especially in the subgroup with total WBC count >10 × 10^9^/L, neutral granular cell count >7 × 10^9^/L, and NLR >1.5. Furthermore, accumulating evidence confirmed that delayed re-endothelialization (49) and endothelial dysfunction (50) play major roles in the development of ISR and are significant predictors of ISR after stent implantation. Among patients with restenosis of the stent, insulin resistance was an established and acknowledged contributory element. The higher incidence of MACEs was correlated with endothelial dysfunction and dysregulated glucose homeostasis, which play a significant role in restenosis (49). Therefore, delayed re-endothelialization and endothelial dysfunction are potential mechanisms in the progression of ISR under the setting of high glycemic variability (50). Previous studies have reported (51) that endothelial vasomotor function in the systemic artery tree is significantly related to the pathobiological process of ISR by suppressing the proliferation of smooth muscle and inhibiting intimal hyperplasia. Endothelial vasomotor function has been shown to reflect nitric oxide-mediated dilation (52). Furthermore, asymmetric dimethylarginine has been shown to be correlated with the pathogenesis of atherosclerosis and endothelial dysfunction (51). A previous study (53) revealed that serum soluble triggering receptor expressed on myeloid cells-1 (sTREM-1) level, which is considerably affected by DM, is a predictive biomarker of ISR and an important mediator of migration, cellular inflammation, vascular smooth muscle cell proliferation, and sTREM-1 concentration. A high ISR rate may be related to dyslipidemia in DM, mainly due to increased remnant-like particle cholesterol, which is identified as lipoproteins rich in triglycerides, and in the fasting state, very low density lipoproteins are major components (54). Estimated GFR <60 ml/min·m^2^, a pre-clinical sign of end-stage renal disease, was a strong independent predictor of documented poor clinical outcome among patients with acute MI undergoing successful PCI (55). Due to lipid metabolic disturbance (56), raised inflammation and oxidative stress (57), elevated the level of serum of homocysteine (58), coagulation and endothelial dysfunction (59), jeopardized homeostasis of calcium phosphate (60), renal insufficiency lead to poor clinical outcome. Levey et al. (61) suggested that the most reliable method to estimate GFR was MDRD equation, which takes gender and age into consideration. In the present study, we have used the equation of MDRD to translate into a reliable assessment of kidney function.

### Limitations

study had some limitations. First, we retrospectively collected the clinical data on definite ST in patients who underwent primary PCI, as reported by site investigators in this study. Furthermore, the trial was conducted in a single center in China. Therefore, we cannot exclude geographical variations in PCI practice outside China or in higher-volume centers. Third, we did not enroll patients with ST in terms of probability, possibility, or secondary to chance, which may have led to an underestimation of the actual ST incidence. However, ST was an endpoint pre-specified according to the definitions of ARC (26). All ST events were adjudicated independently by a blinded clinical events committee according to established criteria, and the incidence of definite ST continued to diverge between the two investigated devices for up to 5 years, which would render a chance finding unlikely. Thus, it is necessary to evaluate demographic covariates and the longitudinal management of therapeutic options carefully. Furthermore, the use of ticagrelor/prasugrel was lower than that of clopidogrel, which could have influenced the clinical outcomes and should therefore be considered a study limitation.

## Conclusion

In conclusion, the study found that DM patients with DNLs have a higher incidence of composite clinical outcomes than their non-DM counterparts. Furthermore, compared with patients with DNL, patients with LST/VLST lesions had more long-term composite clinical outcome events. Thus, LST/VLST lesions are critical problems after coronary stenting, particularly among DM patients. Stronger antithrombotic therapy may help to reduce the incidence of ST and improve clinical outcomes after PCI in patients with type 2 DM.

## Data Availability Statement

The datasets used and/or analyzed during this study are available from the corresponding author on reasonable request.

## Ethics Statement

This study was conducted according to the principles which were outlined in the declaration of Helsinki and has been approved by the Ethics Committee of Fuwai Hospital. All study subjects have provided written informed consent. Written informed consent for publication was obtained from all participants.

## Author Contributions

HY, XZ, JZ, YT, RC, YW, CL, PZ, ZS, JLi, YC, LS, and HZ: substantial contributions to conception and design, data acquisition, or data analysis and interpretation. HY, XZ, JLa, XY, JZ, RC, YW, YT, CL, PZ, ZS, JLi, YC, LS, and HZ: drafting the article or critically revising it for important intellectual content, final approval of the version to be published, and agreement to be accountable for all aspects of the work in ensuring that questions related to the accuracy or integrity of the work are appropriately investigated and resolved. All authors contributed to the article and approved the submitted version.

## Conflict of Interest

The authors declare that the research was conducted in the absence of any commercial or financial relationships that could be construed as a potential conflict of interest.
